# Molecular profiling of endometrial cancer in Martinique reveals frequent *CCNE1* amplification in poor prognosis tumors

**DOI:** 10.1038/s41598-025-24580-w

**Published:** 2025-11-19

**Authors:** Taina Labeau, Jean-Samuel Loger, Mehdi Jean-Laurent, Quentin Hurlot, Cloé Jean-Laurent, Ludivine Chevallier, Sabrina Pennont, Sarah Lise, Sarah Amari, Judicaelle Montlouis-Calixte, Odile Béra, Alexis Vallard, Heriniaina Randriamiarisoa, Emeline Colomba, Régine Marlin

**Affiliations:** 1 Department of Cancer Molecular Genetics, University Hospital of Martinique, Martinique, France; 2 Department of Cancer Medicine, Institut Gustave Roussy, University of Paris Saclay, Paris, France; 3 Department of Gynecological and Breast Surgery, University Hospital of Martinique, Martinique, France; 4 Department of Pathology, University Hospital of Martinique, Martinique, France; 5 Martinique Regional Oncology Platform, University Hospital of Martinique, Martinique, France

**Keywords:** Endometrial cancer, *CCNE1* amplification, Ethnic disparities, African descent, Cancer, Gynaecological cancer, Biomarkers, Predictive markers

## Abstract

**Purpose:**

In Martinique, there is an unmet need in EC management. Although the incidence rate is similar to that in mainland France, the mortality rate is higher, potentially due to the over-incidence of high-grade tumors. Recently, we reported *CCNE1* amplification in 70% of these tumors which have a poor prognosis. This alteration has been observed in non-endometrioid subtypes of African American women. To elucidate the over-incidence of poor prognosis EC in Martinique, we aim to describe molecular profiles especially *CCNE1* amplification of a cohort of all patient diagnosed between January 2023 and June 2024.

**Methods:**

*CCNE1* amplification status was determined using digital PCR. We also performed the analysis of *POLE*, MMR and *TP53* to classified tumors in current molecular classification. A total of 55 patients were included in our study, revealing a different distribution of biomarkers than described in the literature.

**Results:**

We reported an over-incidence of *TP53* mutations and *CCNE1* amplification. Conversely, the prevalence of *POLE* mutations was lower. In this study, non-endometrioid subtypes were particularly associated with *CCNE1* amplification that is consistent with previous studies. The molecular classification observed in our cohort is consistent with findings in populations of African descent. The higher *CCNE1* amplification rate mirrors those seen in these populations.

**Conclusion:**

Given the ethnic origin of the Martinique population, these data suggest that *CCNE1* amplification may be linked to African genetic heritage and could explain the over-incidence of non-endometrioid subtypes in these populations. Furthermore, our study contributes to addressing racial disparities in endometrial cancer outcomes by providing crucial insights into the genetic factors that may influence the prognosis of African-descended populations.

**Supplementary Information:**

The online version contains supplementary material available at 10.1038/s41598-025-24580-w.

## Introduction

In Martinique, managing endometrial cancer (EC) presents a significant challenge for healthcare professionals. According to data from the Martinique Cancer Registry (MCR), approximately thirty new cases are reported annually in a population of around 380,000. The average standardized incidence is estimated at 7.8 per 100,000^1^. While this incidence is comparable to that in mainland France (10.5 per 100,000)^2^, the mortality rate reported is higher. Indeed, the standardized net survival rate (SNS) five years after diagnosis in Martinique is 50%, compared with 74% in mainland France^3^. This higher mortality could be explained by the over-incidence of non-endometrioid tumors such as uterine papillary serous carcinoma (UPSC), carcinosarcoma (UCS), and uterine clear cell carcinoma (UCCC) that we have reported^4^. These observations highlight a racial disparity in EC between the population of Martinique, which is predominantly of African descent (> 90%), and the population of mainland France. A similar racial disparity has been observed in African-American women, who are more likely to present with advanced disease and have poorer outcomes at all stages of high-grade endometrial cancer, such as serous carcinoma^5–9^. The mortality rate is higher among women of African descent than among Caucasian women despite similar incidence rates.^5,10–13^. Genetic or molecular differences may contribute to the development of these tumors. A recent study comparing the molecular characteristics of endometrial tumors in patients of African descent and Caucasian patients revealed distinct drivers with different therapeutic implications^5^. *CCNE1* amplification appears to be the primary biomarker associated with non-endometrioid tumors in populations of African descent. More recently, we conducted an exome-wide study on the molecular characterization of *TP53*-mutated high-grade endometrial tumors diagnosed in Martinique, which also detected *CCNE1* amplification in 70% of tumors analyzed^14^. *CCNE1* amplification has been described in EC, particularly in high-grade endometrioid and non-endometrioid cancer^5,15^. However, it remains unclear whether this alteration reflects the poor prognosis typically associated with high-grade histology or if it serves as an independent prognostic factor. In UCS carcinoma, Fluorescence in Situ Hybridization (FISH) analysis reveals amplification in about 45% of tumors^7,16^. Furthermore, according to literature data and the TCGA database, *CCNE1* amplification appears to be enriched in non-endometrioid carcinoma in patients of African descent^5,7^. This alteration is responsible for replication stress with widespread copy number gains and losses, a form of genomic instability known to be associated with poor prognosis. *CCNE1* encodes the cyclin E1 protein, which complexes with CDK2 to promote cell cycle progression from the G1 phase to the S phase^17^. Overexpression of cyclin E1 leads to premature entry into the S phase, resulting in increased stress at replication forks and double-strand DNA breaks (DSBs)^17,18^. Today, it is well-established that CCNE1 is an oncogene involved in the oncogenesis of several tumor types^19^, particularly high-grade gynecological tumors^5,7,20,21^. Interestingly enough, the involvement of CCNE1 in the oncogenesis of these tumors has led to the development of targeted therapies that promise better outcomes for patients. These therapies are based on the hypothesis that CCNE1 overexpression increases sensitivity to replication checkpoint inhibitors^19,22^. Indeed, overexpression of CCNE1 results in the loss of the G1-S checkpoint, increasing dependence on the S phase at the G2-M cell cycle checkpoint to ensure cell survival^23–25^. Increased sensitivity of ATR, CHK1, and WEE1, which were checkpoint transitions in the S and G2/M phases, has been demonstrated in cells with *CCNE1* amplification^22,26,27^. These studies showed that EC cells overexpressing CCNE1 could be selectively eliminated, offering therapeutic hope for patients with endometrial tumors carrying *CCNE1* amplification.

The Cancer Genome Atlas (TCGA) landmark study has classified EC into four distinct molecular groups: (i) POLE-ultra-mutated (POLEmut) ECs, (ii) microsatellite instability (MSI/hypermutated) ECs, (iii) serous-like/copy number high (p53-abn) ECs and (iv) the copy number low/microsatellite stable ECs or No Specific Molecular Profil (NSMP)^28^, each with clearly established prognostic value. Currently, despite the identification of a molecular profile, therapeutic strategies are not specific to each subgroup. Indeed, several trials addressing the question of therapies according to each subgroup is on going^29^.

Given the histological and molecular specificities described in ECs of African descent, and previous finding of a strong association between *CCNE1* amplification and high-grade endometrial tumors, we designed a comprehensive study including all endometrial cancers diagnosed in Martinique over an 18-month period. Our objective was to establish both the histological and molecular landscape of endometrial cancers in this population, and to confirm whether *CCNE1* amplification is specifically associated with the over-represented high-grade subgroups.

## Results

### Current endometrial cancer molecular subtypes

**Fig. 1:**
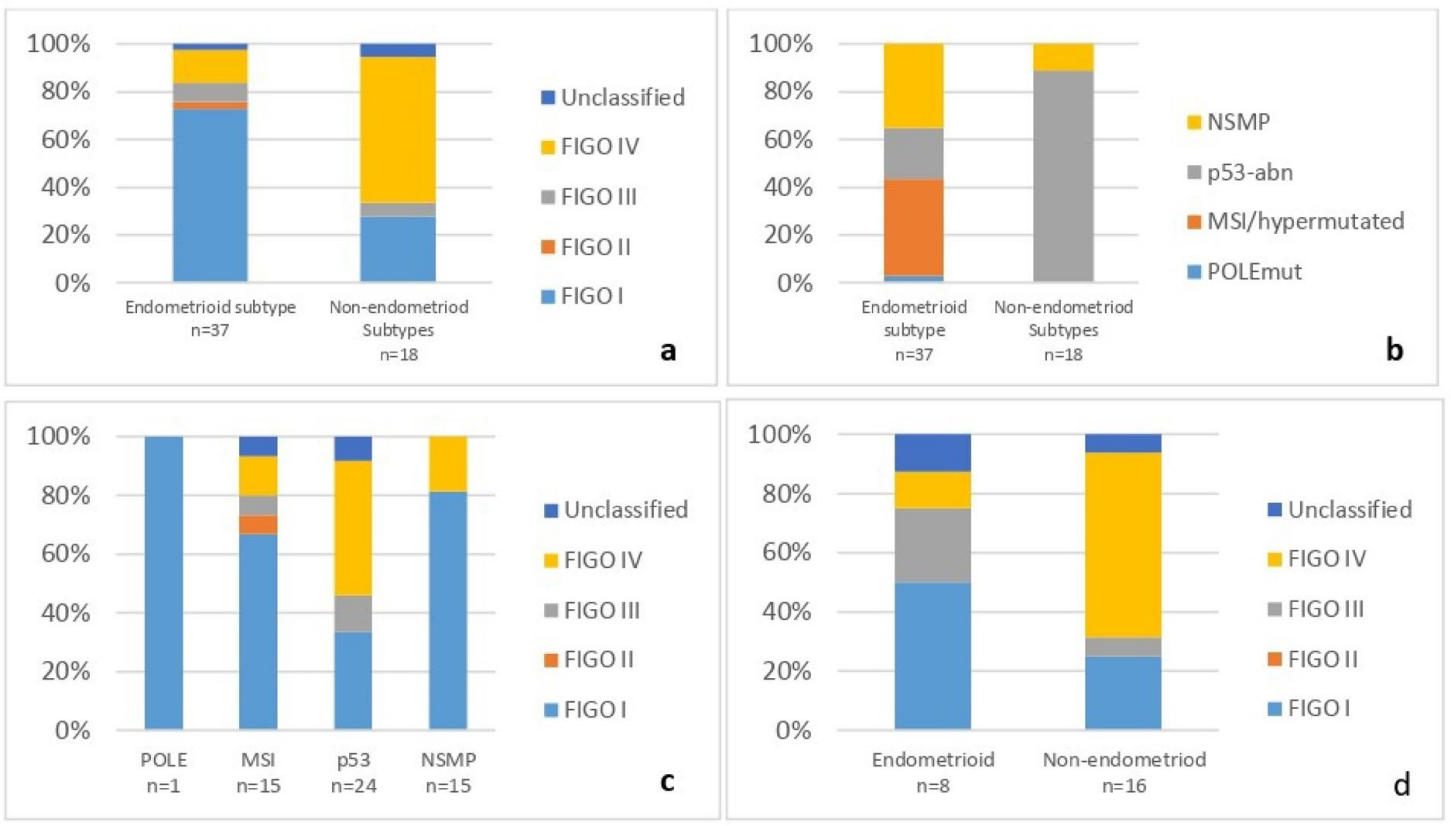
Histologic, clinical and molecular subtypes distribution of endometrial cancer in Martinique.** a**, Distribution of FIGO stage in endometrioid and non-endometrioid subtypes. **b**, Distribution of molecular groups in endometrioid and non-endometrioid subtypes. **c**, Distribution of FIGO stage in molecular groups. **d**, Distribution of FIGO stage in endometrioid and non-endometrioid subtypes classified p53-abn.

Patient enrollment included 55 EC subjects over 18 months: 67.2% (37/55) had endometrioid carcinoma, and 32.7% (18/55) had UPSC, UCS, or mixed types. The median age at diagnosis was 69 years [range: 48–91]. The majority of diagnosed tumors were at a localized FIGO stage I and II (59.9% 33/55), and most of these localized stage tumors were of the endometrioid type (50.9%) (27/55) (Table 1 and Fig. 1a). Additionally, 29.1% (16/55) of all patients had metastases at diagnosis. Over 61.1% (11/18) of non-endometrioid tumors were diagnosed with metastases, compared to only 13.5% (5/37) of endometrioid tumors (Table 1 and Fig. 1a).

**Table 1:** Tumor and patient characteristics.

	All n = 55
Age at diagnosis (Mean)	69 [50–90]
FIGO stage	
*FIGO I*	32 (58.1%)
*FIGO II*	1 (1.8%)
*FIGO III*	4 (7.3%)
*FIGO IV*	16 (29.1%)
*Missing*	2 (3.6%)
Histology subgroups	
Serous	13 (23.6%)
Endometriod	37 (67,3%)
Carcinosarcome	2 (3.6%)
Clear cell	1 (1.8%)
Mixte	2 (3.6%)

**Fig. 2:**
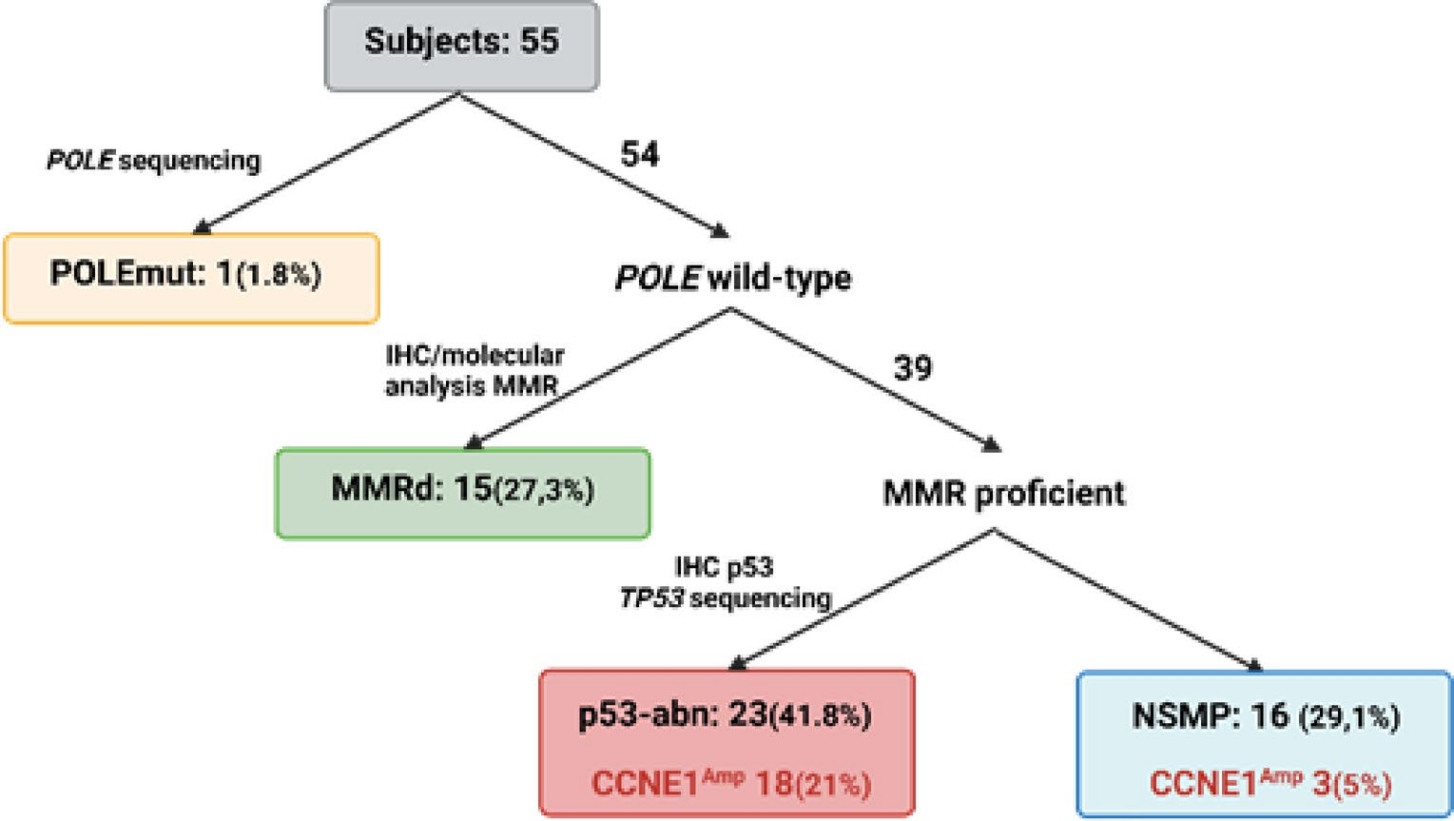
Molecular distribution of endometrial carcinomas according to the diagnostic algorithm, with additional *CCNE1* status. Among the 55 patients included, 1 tumor was classified as POLE-mutated (1.81%), 15 as MMRd (27.27%), 24 as p53-abn (43.63%) of which 12 harbored *CCNE1* amplification, and 15 as NSMP (27.27%) of which 3 harbored *CCNE1* amplification.

The distribution of the current molecular classification was as follows: POLEmut subtype 1.8% (1/55), MSI/hypermutated subtype 27.27% (15/55), p53-abn subtype 43.63% (24/55), and NSMP 27.27.1% (15/55) (Table 2 and Fig. 2). In line with literature data, most tumors with *TP53* mutations were high-grade and diagnosed at an advanced stage (Fig. 1b and 1c). Of these, 66.66% were non-endometrioid, and 33.33% (8/24) were endometrioid (Fig. 1b). More than half of the p53-abn endometrioid tumors were diagnosed at an early stage, in contrast to the non-endometrioid p53-abn subtypes, which mainly were diagnosed at an advanced FIGO stage IV (Fig. 1d). Among NSMP tumors, 13/15 (87%) belonged to the endometrioid subtype. Among the MSI/hypermutated subtype tumors, 13/15 (87%) showed a loss of MLH1/PMS2 proteins. Only one tumor did not show hypermethylation of the *MLH1* promoter, suggesting Lynch syndrome. Additionally, two tumors showed isolated loss of expression—one of PMS2 and the other of MLH1. A constitutional *MLH1* mutation was identified in the patient with isolated MLH1 loss, while for the patient with PMS2 loss, constitutional analysis of the *PMS2* gene could not be performed.

**Table 2:** Molecular features.

	All n (%)	Endometrioid subtype n (%)	No-endometriod Subtypes n (%)
*POLE*			
*No mutated*	54 (98.2)	36 (97,3)	18 (100)
*Mutated*	1 (1.8)	1 (2.7)	0 (0)
MMR			
*Proficient*	40 (72.7)	22 (59.5)	18 (100)
*Deficient*	15 (27.3)	15 (40.5)	0 (0)
*TP53*			
*No mutated*	31 (56.4)	29 (78.4)	2 (11.1)
*Mutated*	23 (41.8)	8 (21.6)	15 (88.9)
NSMP	16 (29,1)	14 (37.8)	2 (11.1)
*CCNE1*			
*CN* < *3*	32 (58.2)	29 (78.4)	3 (16.7)
*CN 3–5*	12 (21.8)	2 (5.4)	10 (55.6)
*CN* > *5*	3 (5.5)	1 (2.7)	2 (11.1)
*Unknown*	8 (14.6)	5 (13.5)	3 (16.7)

### CCNE1 analysis

To validate the digital PCR (dPCR) assay, we first used 17 tumor samples from our previous study (reference 14), in which *CCNE1* copy number status had been determined by whole-exome sequencing (WES). The results were fully concordant between WES and dPCR, confirming the robustness of the assay.

**Fig. 3:**
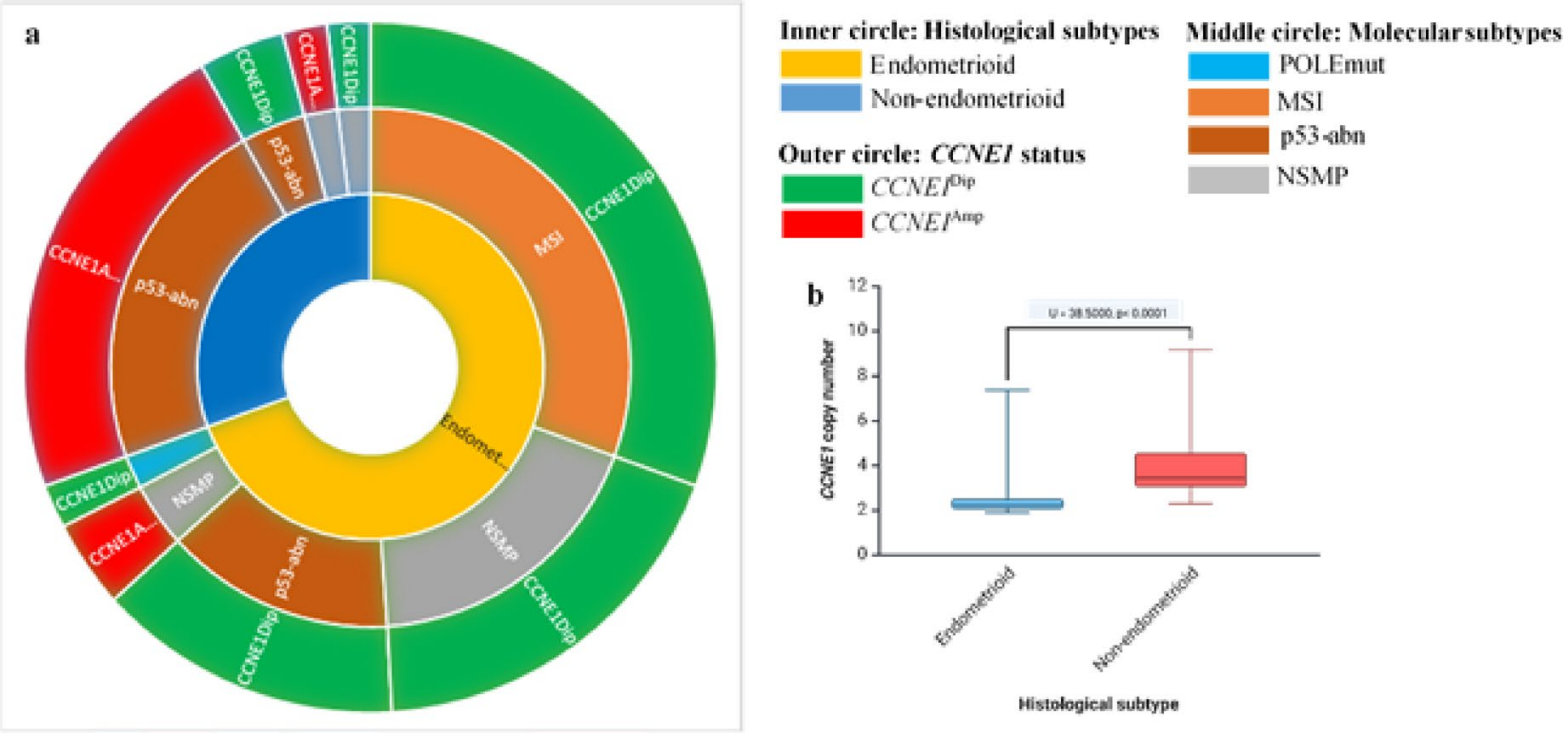
*CCNE1* copy number. **a,** Association between histologic types, molecular subtypes and *CCNE1* status of endometrial cancers. **b,** BoxPlot showin**g** *CCNE1* copy number by histologic subtype.

**Fig. 4:**
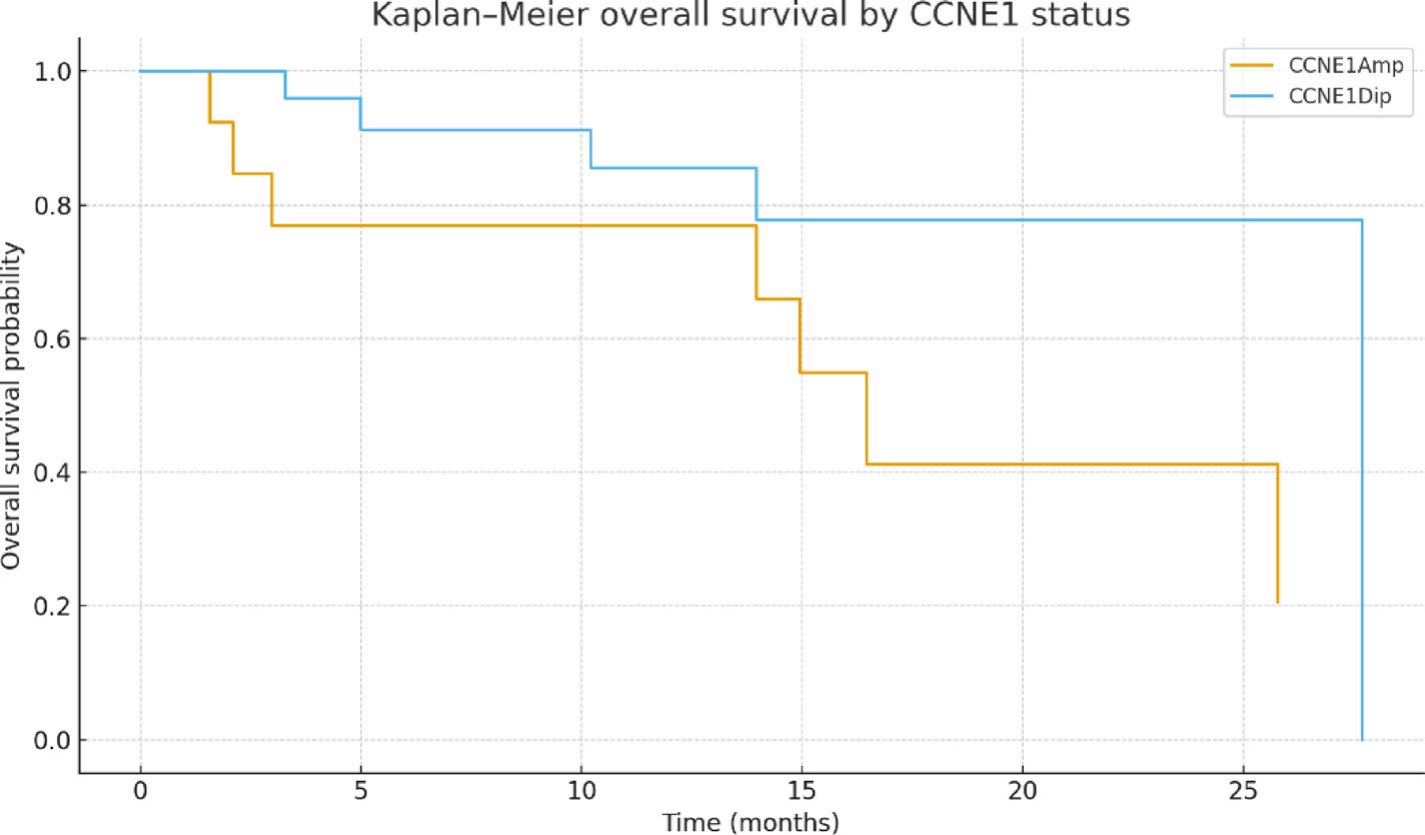
Kaplan–Meier overall survival according to *CCNE1* status. Kaplan–Meier curves comparing overall survival in patients with *CCNE1*-amplified (CCNE1Amp, n = 15) and non-amplified tumors (CCNE1Dip, n = 32). Median overall survival was shorter in the *CCNE1*-amplified group (16.5 months) compared with the non-amplified group (27.7 months). Twelve-month OS was 0.77 versus 0.86, and 24-month OS was 0.41 versus 0.78, respectively. The difference did not reach statistical significance (log-rank test, p = 0.148).

In the present cohort of 55 newly diagnosed endometrial carcinomas, *CCNE1* status was assessed exclusively by dPCR. Results were obtained for 47 tumors; no additional material was available in five cases, and three analyses were non-contributory. Among the 47 tumors analyzed, 32 had a copy number < 3. Notably, 66% (12/18) of non-endometrioid tumors were amplified, compared to only 8% (3/37) of endometrioid subtypes (Fig. 3). This amplification was significantly associated with non-endometrioid subtypes (Mann–Whitney U = 38.5000, p < 0.0001). Further analysis showed that 50% (12/24) of tumors with *TP53* mutations also harbored *CCNE1* amplification, all of which were non-endometrioid. Conversely, *TP53*-mutated tumors without *CCNE1* amplification were primarily endometrioid. Interestingly, three tumors (18%) within the NSMP molecular subgroup carried *CCNE1* amplification. Among these, two were diagnosed at an advanced stage, presenting with peritoneal and digestive metastases. Importantly, the distribution of FIGO stages differed according to *CCNE1* status: 67% (10/15) of *CCNE1*-amplified tumors were diagnosed at advanced stages (FIGO III–IV), compared with only 17% (6/32) of non-amplified tumors (Chi-square test, p = 0.0017; Fisher’s exact test, p = 0.0017) (Table S1). In addition, overall survival analysis showed shorter median OS in patients with *CCNE1* amplification (16.5 months) compared with non-amplified cases (27.7 months). Twelve-month OS was 0.77 for *CCNE1*-amplified tumors versus 0.86 for non-amplified tumors; at 24 months, OS was 0.41 versus 0.78, respectively (Fig. 4). Although the log-rank test did not reach statistical significance (p = 0.148), likely reflecting limited sample size and number of events, these results are consistent with an association between *CCNE1* amplification and poorer prognosis.

### Genetic ancestry association

**Fig. 5:**
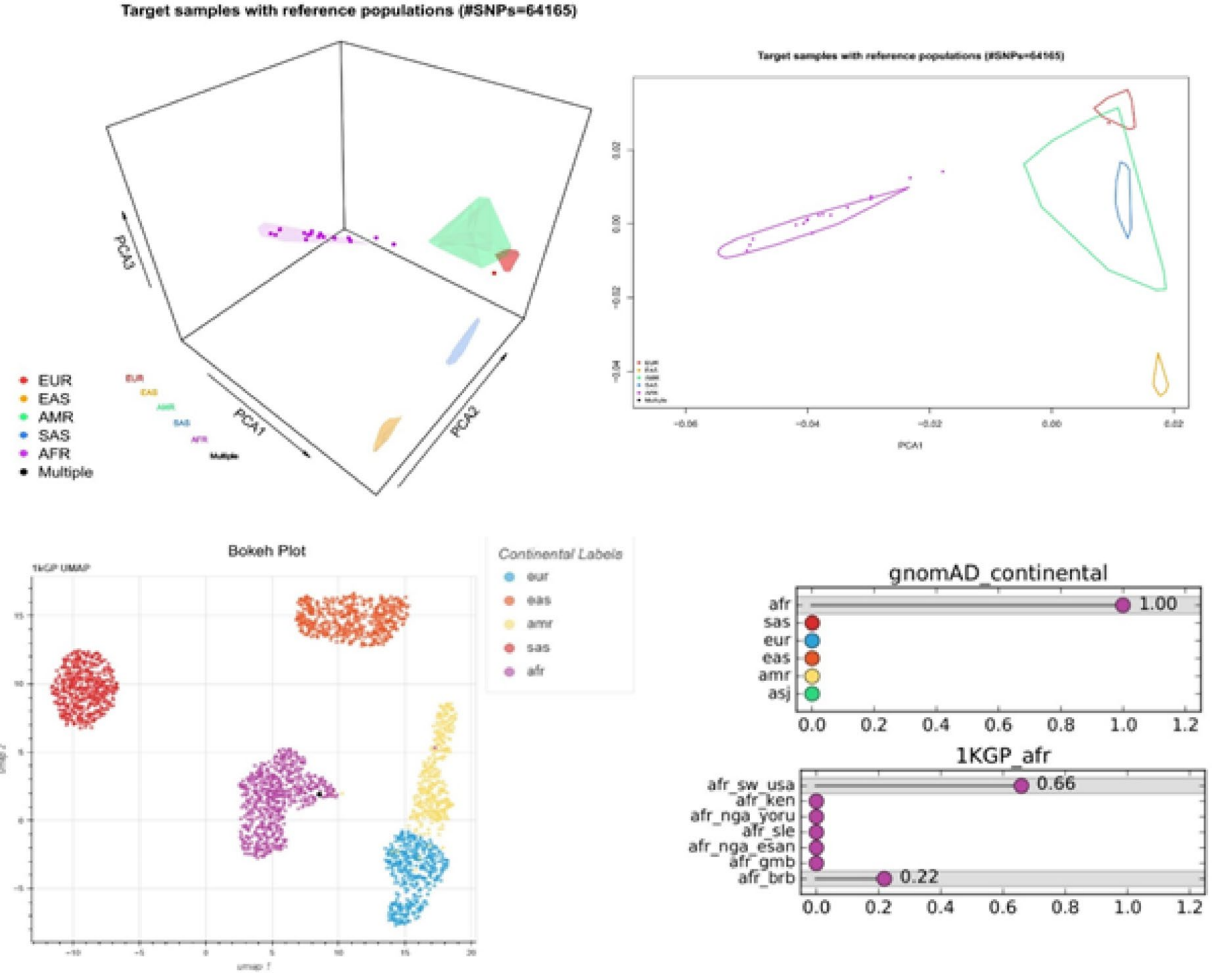
Genetic ancestry of patients endometrial cancer in Martinique. **a,** Output of EthSEQ analysis of 17 subjects. **b,** Output of SNVstory, Single patient presentation.

Most of our patients’ SNPs align with the African population, with only one patient aligning with the European population (Fig. 4a). We compared 64,165 SNPs with polymorphisms from control populations. Multiple correspondence analysis further confirmed our patients’ African genetic heritage (Fig. 3). Additionally, our patients share genetic variants with populations from the African Southwestern USA and African Caribbean in Barbados (Fig. 5).

## Discussion

We reported for the first time the molecular profiles of EC patients diagnosed over 18 months, representing a homogeneous cohort from diversity. More than 35% (20/55) of the patients are diagnosed with advanded stage III-IV. Moreover, the histology was unusual with 33% (18/55) of non-endometrioid subtype (Table 1). These findings confirm our previous study, which reported an over-incidence of aggressive forms in our population^30^. Generally, early-stage disease accounts for 80% of all tumors^31^. Identifying the molecular mechanisms responsible for the initiation and progression of advanced tumors, including potential therapeutic targets, is crucial for improving patient management in Martinique, especially given the racial disparities observed. We observed a histological and molecular distribution in our cohort that differs from what is typically described in Caucasian patients, showing similarities to the distribution found in the African descent cohort analyzed in the Weigelt study^5^ (Fig. 1a and 2), though the sample size is small. Specifically, our cohort has a significantly higher prevalence of non-endometrioid subtypes associated with a higher prevalence of *TP53* mutations and *CCNE1* amplification. These current results are consistent with our previous findings, further supporting the link between *CCNE1* amplification and poor prognosis in high-grade ECs. Nearly a quarter of the tumors exhibited *CCNE1* amplification (Fig. 3). This biomarker, previously linked to high-grade tumors^7,20,21,32^, is more frequently identified in EC from African women (20%) compared to Caucasian women (10%) according to TCGA database and literature data^5,7^. The *POLE* mutation rate (1.8%) in our cohort is also similar to that observed in populations of African descent and lower than the rate found among white American women (5.8%)^5^. Our genetic ancestry analysis confirmed that our patients have African genetic origins (Fig. 5a and 5b), sharing the same genetic heritage as those from African ancestry in the Southwestern USA and the African Caribbean in Barbados (Fig. 5). This finding is important to contextualize the molecular alterations observed, as it suggests that clinical outcomes and therapeutic responses in our population may resemble those of African-American women more closely than those of Caucasian cohorts. Although Martinique benefits from the French healthcare system, these results underscore the need to promote the inclusion of Afro-Caribbean patients in clinical trials to ensure that management strategies adequately reflect their genetic and clinical specificities. The unique migratory history of African Americans and Caribbeans appears to contribute to distinct genetic characteristics, suggesting that these populations might be considered separate ethnic subgroups from other African populations. Understanding the specificities of this subgroup will enhance our comprehension of risk factors for cancers with a more aggressive phenotype. The identification of *CCNE1* amplification as a primary biomarker for high-grade EC in our population^14^ consistent with reports in African American women^5^ suggests that it could represent a relevant marker for future studies aiming to improve risk stratification. Notably, our latest results validate our initial findings, with 66% of non-endometrioid subtypes harboring *CCNE1* amplification compared to 8% of endometrioid tumors, demonstrating a strong association between *CCNE1* amplification and non-endometrioid subtypes (p < 0.0001). Given the poor prognosis of these subtypes and the advanced stage at diagnosis (Fig. 1a), *CCNE1* status analysis may be particularly relevant in individuals of African descent, especially in Martinique. In our cohort, *CCNE1* amplification was enriched in advanced FIGO stages (Supplementary Table 1) and was associated with a trend toward shorter overall survival (Fig. 4), although this did not reach statistical significance. dPCR, being a rapid and cost-effective technique, could represent a promising approach for detecting *CCNE1* amplification, although its potential for routine clinical implementation requires validation in larger studies and clinical trials.

*CCNE1* amplification, which appears to be responsible for the increased incidence of non-endometrioid subtypes in populations of African descent^5,6,8^, could be attributed to African genetic heritage. Further germline studies are needed to validate this hypothesis.

Our results also indicate specificities within the Martinique population that are distinct from those of African descent populations. The prevalence of the MSI/hypermutated subtype in our cohort is similar to that observed in Caucasian cohorts^33–35^, with 27% (15/55) in our study compared to 14% of dMMR tumors detected by Weigelt^5^. This suggests the need for targeted studies to establish a specific management approach for endometrial tumors in Martinique. In our study, dMMR and CCNE1-amplified tumors accounted for more than half of all diagnosed cases, which raises the possibility of targeted therapeutic strategies. While exploratory, these findings support the rationale for further investigation of immunotherapy and cell cycle checkpoint inhibitors in this setting through dedicated clinical trials. Although we did not directly assess TMB or CIN in this study, MSI status was analyzed and provides indirect information on mutational load.

Our study provides insight into the current molecular classification by linking *CCNE1* amplification to non-endometrioid tumors. Interestingly, we also detected *CCNE1* amplification in three tumors within the NSMP molecular subgroup. Although preliminary, this observation suggests that additional oncogenic mechanisms may contribute to this heterogeneous group and warrants further investigation in larger cohorts. Such efforts may also lead to the discovery of other relevant biomarkers with potential therapeutic implications. More than 40% (24/55) of tumors in our study did not reveal the molecular mechanisms involved in oncogenesis, belonging to the NSMP and p53-abn subgroups. Identifying *CCNE1* amplification as a main oncogenic driver in these subgroups supports exploring the involved molecular mechanisms. p53 may not act as an oncogenic driver but may exert selective pressure, providing a growth advantage to cancer cells and leading to their dominance^36^. Most tumors lacking identified oncogenic mechanisms. Further studies are needed to confirm our initial results and identify unknown molecular mechanisms. The Martinique population is particularly well-suited for such investigations, as it offers the possibility of assembling more homogeneous cohorts and better controlling environmental and cultural variables, thereby reducing noise in molecular data and allowing the identification of specific somatic alterations. These efforts are essential to address health disparities in endometrial cancer, as populations of African descent, despite being disproportionately affected by aggressive forms of the disease, remain underrepresented in databases and clinical studies^5,6,9^. To move forward, we believe that a dual strategy is required: on the one hand, comprehensive genomic studies including paired germline and somatic analyses to elucidate oncogenic mechanisms in underrepresented populations, and on the other hand, the implementation of cost-effective assays such as digital PCR for *CCNE1* amplification to enable real-time patient stratification. Ultimately, the integration of these approaches into clinical trials will be crucial to evaluate the true impact of molecular alterations on prognosis and therapeutic response.

## Conclusions

Martinique, a French department benefiting from the same healthcare system as metropolitan France, faces unique challenges in managing endometrial tumors due to the genetic origins of its population. Our study highlights the value of enhanced molecular characterization to better understand tumor initiation and progression in this context. We observed genetic specificities in our cohort that align with those described in populations of African descent. In the era of precision medicine, a deeper understanding of the genetic characteristics of diverse populations is crucial for developing effective prevention and treatment strategies. While our results are exploratory and based on a limited sample size, they underscore the importance of conducting local studies in underrepresented populations, which may ultimately help reduce health disparities and inform future clinical research. Addressing these specificities will require not only local studies but also the active inclusion of Afro-Caribbean patients in clinical trials, in order to reduce health disparities and improve outcomes.

## Methods

***Ethical statement.***

The patients were seen as part of their routine care and provided informed consent for their samples could be used for research purposes. At the time of diagnosis, they provided informed consent for their samples could be used for research purposes. The protocol approved by the institutional review board of university hospital of Martinique (approval # 051). This study followed the ethical guidelines for clinical application, in accordance with the Declaration of Helsinki.

### Patient and control subjects

The patients enrolled in this study were diagnosed with EC between January 2023 and June 2024 in Martinique underwent analysis as part of their routine care. A dedicated pathologist performed the histological classification according to the World Health Organization (WHO) recommendations^37^. Detailed clinical information related to the EC diagnosis was collected, including age at diagnosis, body mass index (BMI), parity, menopausal status, comorbidities (such as hypertension, diabetes, and others), treatment received, histological type, stage at diagnosis (TNM), and FIGO classification.

For assay validation, we used 17 control tumor samples from our previous study^14^, in which *CCNE1* status had been determined by whole-exome sequencing (WES) These samples were re-analyzed by dPCR to confirm concordance between the two methods. We have categorized the samples into the following groups: *CCNE1*^Dip^ [CN] < 3 and *CCNE1*^Amp^ [CN] > 3.

### Current Endometrial Cancer Molecular Subtypes

Immunohistochemistry (IHC) and molecular analysis determined the current molecular subtypes. A pathologist selected a representative formalin-fixed paraffin-embedded (FFPE) tumor block for each case.

#### Immunohistochemical testing

All IHC analyses were performed on the Ventana Benchmark autostaining system using the following mouse monoclonal antibodies: DO-7 (0.5 µg/ml) for p53, M1 (1 µg/ml) for MLH1, A16-4 (1 µg/ml) for PMS2, G219-1129 (20 µg/ml) for MSH2, and SP93 (1 µg/ml) for MSH6. The slides were pretreated in CC1-COURT buffer for p53 IHC and CC1-LONG for 92 min for MMR IHC. Antibodies were incubated at 37 °C for 60 min and then detected using the ULTRAVIEW-DAB system (Roche/Ventana Medical Systems).

For p53 IHC, cases were categorized into one of three groups. Wild-type p53 expression was defined as nuclear staining of variable intensity in 1–80% of the tumor. Null expression was characterized by the absence of p53 nuclear staining with positive internal control, while overexpression was identified by uniform and intense nuclear staining in at least 80% of tumor cell nuclei. It is important to note that, for the purposes of this study, ‘ambiguous’ p53 expression was not considered a category, ensuring that all cases were assigned one of the three.

Pathologists assessed MMR protein expression following established pathological guidelines^38–40^. Staining was first validated using positive control nuclei. MMR protein expression in tumor cell nuclei was then evaluated, emphasizing identifying conserved or lost expression for each MLH1, MSH2, MSH6, and PMS2 protein. Adjacent normal epithelial cells, stromal cells, and inflammatory cells with intact nuclear staining were internal positive controls.

#### Microsatellite instability analysis and MLH1 promotor DNA methylation

MSI was assessed by amplifying the Pentaplex mononucleotide repeat panel (BAT-25, BAT-26, NR-21, NR-22, and NR-24). The fragments are analyzed on an ABI PRISM 3500 genetic analyzer (Applied Biosystems®, Foster City, CA, USA), as previously described^41^. From extracted tumor DNA, MSI (dMMR) was defined by the instability of at least two microsatellite markers. dMMR status was compared to MMR IHC. *MLH1* promotor DNA methylation was analyzed using an EZ DNA methylation-gold kit (Zymo research, Proteigene) and PyroMark Q24 CpG MLH1® Kit (Qiagen) in accordance with the manufacturer’s instructions.

#### Genetic analysis

Tumor sequencing was performed using a commercial SophiaGenetics solution, including *POL-E* and *TP53* genes. This panel of 22 genes is commonly used in the laboratory. The libraries were sequenced on a MiSeq platform (Illumina, San Diego, California, USA), and the bioinformatics pipeline was executed using SOPHiA DDM software. Only pathogenic somatic missense mutations within the DNA polymerase epsilon (POLE) exonuclease domain defining the important subtype of ultramutated tumors POLE‐ultramutated are retained^42^. For *TP53* mutations, only nonsense, frameshift, and missense variants described as pathogenic according to ClinVar were reported.

### CCNE1 analysis

In the present study, all 55 newly diagnosed tumors were analyzed exclusively by digital PCR (dPCR) using the QuantStudio 3D Digital PCR System (Life Technologies, Waltham, MA) to determine the *CCNE1* copy number (*CCNE1* CN) as described previously^43^. We used three pairs of FAM-labeled primers targeting *CCNE1* designed by Thermofischer (Assay HS01813172; HS02208561 and Hs02252990). The ABY-labeled probe for RNase P (TaqMan® RNASEP Assay) targeting the single exon region of the *RPPH1* gene was used as a reference for two copies. All probes had either QSY or IBRQ dark quenchers on their 3′ ends. All samples were analyzed in triplicate. CN value was determined using the Absolute Q Analysis Sofware, which automatically calculates the optimal positive/negative threshold for each target. For each unit, the software displays the total viable partition count and the positive partition count.

First, we validate the dPCR using the control samples whose *CCNE1* status had been identified by WES^14^. All tumors were re-analyzed by dPCR. The tumors were then classified according to the same categories as the control samples: *CCNE1*^Dip^ and *CCNE1*^Amp^. The Mann–Whitney U test was applied to compare the CN value between endometrioid and non-endometrioid subtypes.

### Statistical analysis

Associations between categorical variables (e.g., FIGO stage and *CCNE1* status) were assessed using the Chi-square test or Fisher’s exact test when appropriate. Overall survival (OS) was defined as the time from diagnosis to death from any cause or last follow-up. Survival curves were estimated using the Kaplan–Meier method, and differences between groups were compared with the log-rank test. Median survival times and survival rates at 12 and 24 months were reported. Statistical significance was set at p < 0.05.

### Genetic ancestry association

We evaluated the ethnic origin of the patients based on the germline WES of control subjects^14^. From this cohort, we identified *CCNE1* amplification in 70% of the tumors. This cohort is representative of the Martinique population. Seventeen patients were included in this analysis. We utilized two computational tools to estimate the ancestry of individuals using NGS data: EthSEQ^44^ and SNVstory^45^. EthSEQ analyzes Single Nucleotide Polymorphism (SNP) genotypes derived from WES to determine an individual’s genetic background. SNP genotypes were extracted from sequencing alignment files in BAM format for our analysis. Ancestry was inferred using a reference model with a standard refinement process based on Principal Component Analysis (PCA).

In addition to EthSEQ, we used SNVstory, a tool built upon three independent machine learning models to accurately infer sub-continental ancestry. SNVstory includes a feature-importance scheme, unique among open-source ancestry tools, which tracks the ancestral signal of specific genes or loci. This tool demonstrated the capability to estimate ancestry from 36 different populations with high accuracy.

## Supplementary Information

Below is the link to the electronic supplementary material.

Supplementary Material 1

## Data Availability

Due to ethical and confidentiality considerations associated with patient care data, the raw sequencing data are not publicly available. However, these data may be shared upon request to the authors, subject to appropriate approval and the signing of a data use agreement. Data access requests should be directed to regine.marlin@chu-martinique.fr, and each request will be reviewed within 2–4 weeks. Data use will be restricted to the purposes specified in the agreement and must comply with relevant ethical and regulatory requirements.
